# Vertebral Artery Occlusion With Ipsilateral Weakness Due to Ischemic Stroke at the Medulla–Cervical Spinal Cord Junction: A Case Report

**DOI:** 10.1002/ccr3.72625

**Published:** 2026-04-28

**Authors:** Peter Felton, Yugant Khand, Mohammad Gharavi, Ram Saha

**Affiliations:** ^1^ Department of Neurology Virginia Commonwealth University Richmond Virginia USA; ^2^ Nepalese Army Institute of Health Sciences ‐ College of Medicine Kathmandu Nepal; ^3^ Department of Radiology Virginia Commonwealth University Richmond Virginia USA

**Keywords:** case report, medullary infarction, neuroanatomy, posterior circulation stroke, pyramidal decussation, vertebral artery occlusion

## Abstract

Posterior circulation strokes can present with diverse neurological deficits, some of which challenge conventional localization principles. We report a case of a 74‐year‐old male with a history of prior right medullary infarct who presented with recurrent symptoms and was found to have a new medullary acute ischemic stroke in the setting of chronic right vertebral artery occlusion. Notably, the patient exhibited ipsilateral limb weakness, a finding attributable to involvement near the pyramidal decussation at the medulla–spinal cord junction. This case underscores the importance of detailed neuroanatomical understanding in vertebrobasilar ischemia and highlights the diagnostic complexity of brainstem strokes.

## Key Clinical Message

Vertebral artery occlusion can result in recurrent ischemia at the medulla–cervical junction, producing ipsilateral weakness due to involvement at or just caudal to the pyramidal decussation. Careful correlation of neuroimaging with neuroanatomical principles is essential to avoid diagnostic delays.

## 1 Introduction

Posterior circulation strokes account for nearly one‐quarter of ischemic strokes but can present with diverse and atypical neurological deficits, often complicating timely diagnosis and localization. This variability is particularly pronounced due to the complex anatomy and perfusion patterns of the brainstem and cerebellum [1]. Classic medial medullary syndrome produces contralateral hemiparesis from corticospinal tract involvement above the pyramidal decussation. In contrast, lesions at or just caudal to the decussation can result in ipsilateral weakness, a pattern that may be misinterpreted as peripheral or spinal pathology [2, 3]. Rare entities of lateral medullary infarction such as Opalski syndrome and variants of Wallenberg syndrome reinforce this clinico‐anatomical paradox, where the lesion's involvement relative to the decussation determines motor laterality [2, 3]. Vertebral artery pathology, including atherosclerotic occlusion, fibromuscular dysplasia dissection, and dolichoectasia, is a recognized cause of medullary infarction [4]. However, detailed clinico‐radiological correlation of cases involving the medulla–spinal cord junction, where the vertebral artery transitions to the anterior spinal artery, remains limited. We present a case of recurrent medullary infarction with ipsilateral hemiparesis due to chronic vertebral artery occlusion, with imaging and clinical findings that illustrate the neuroanatomical basis of this presentation. This report aims to expand the understanding of decussation level lesions and their diagnostic implications in acute stroke evaluation.

## 2 Case Presentation

### 2.1 Case History/Examination

A 74‐year‐old male with a history of right medullary stroke (June 2024) on aspirin 325 mg daily; poorly controlled type 2 diabetes mellitus on insulin; hypertension on hydrochlorothiazide, hydralazine, amlodipine; hyperlipidemia on rosuvastatin 20 mg daily; coronary artery disease on isosorbide dinitrate; chronic kidney disease; and prostate cancer presented to our emergency department for evaluation of new neurological symptoms. The patient reported that on October 19, 2024, around 9–10 PM, he experienced sudden‐onset slurred speech, right‐sided facial droop, difficulty walking, and weakness and numbness of the right arm and leg. He reported being at his baseline a few minutes prior to onset. He had consumed two beers prior to symptom onset and initially attributed his symptoms to alcohol, delaying medical attention.

### 2.2 Differentials, Investigation, Treatment

**FIGURE 1:**
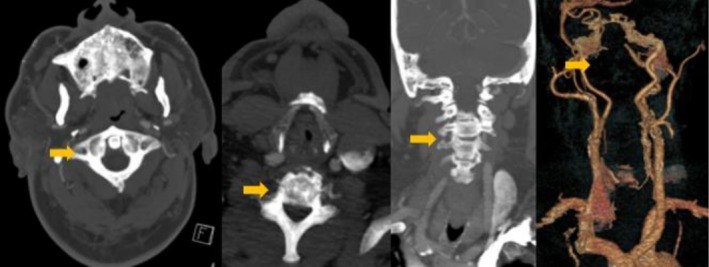
CTA head and neck.

At the outside hospital, computed tomography (CT) Head, which was negative for any hemorrhage or hypodensity, and computed tomography angiography (CTA) of Head and Neck revealed a well‐known vertebral artery occlusion and diffuse atherosclerosis (Figure 1). He was outside the thrombolytic window and was transferred to our hospital for further evaluation. Differentials of cervical myelopathy, lacunar infarct, and peripheral neuropathy were excluded based on neuroimaging.

### 2.3 Outcome and Follow‐Up

**FIGURE 2:**
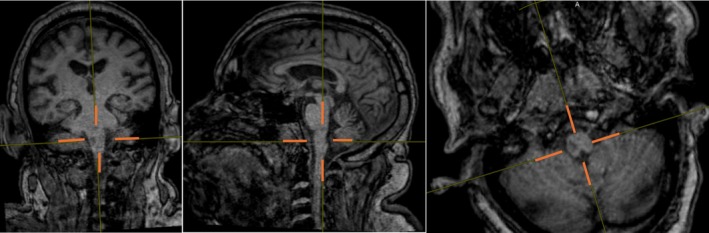
Perpendicular to the axial MRI brain T1 slices displaying the infarct involvement of the pyramidal decussation at the medulla–spinal cord junction.

**FIGURE 3:**
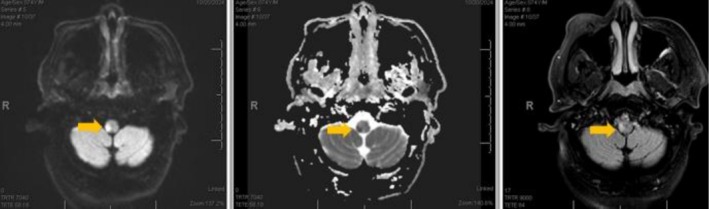
MRI brain without contrast: DWI (left), ADC (middle), flair (right).

On arrival, his National Institutes of Health Stroke Scale score was 5 (mild dysarthria, right upper extremity ataxia, and subtle right facial weakness). Magnetic resonance imaging (MRI) of the brain demonstrated the infarct involvement of the pyramidal decussation at the medulla–spinal cord junction (Figure 2). MRI brain without contrast showed an acute on chronic right medullary infarct (Figure 3). A provisional diagnosis of Opalski Syndrome was made based on pathognomonic features of right medullary infarct and right‐sided hemiparesis affecting both upper and lower extremities due to right vertebral artery occlusion as the causative vascular lesion. Transthoracic echocardiogram showed concentric left ventricular hypertrophy, an ejection fraction of 70%, and normal left atrium. The patient's neurological deficits improved during hospitalization, though mild right upper extremity dysmetria and right lower extremity weakness persisted at discharge.

## 3 Discussion

This case illustrates a rare but anatomically logical presentation of ipsilateral weakness in brainstem stroke. Though ipsilateral weakness in a brainstem lesion is paradoxical, it can be explained by involvement of corticospinal fibers at or below the pyramidal decussation. The corticospinal tracts decussate at the caudal medulla. Therefore, lesions above the decussations result in contralateral hemiparesis, while lesions at or below this level can disrupt fibers destined for the ipsilateral limbs. In our patient, the infarct likely involved the medulla–spinal cord junction, explaining the ipsilateral motor findings, which underscores the importance of accurate topography of the medullary lesion. The mechanism involves extension of the lateral medullary infarction below the pyramidal decussation, affecting the already‐crossed corticospinal tract fibers, resulting in weakness on the same side as the lesion rather than the contralateral side.

Ipsilateral hemiparesis has been described in Opalski syndrome, where lateral medullary infarction is associated with ipsilateral hemiplegia due to involvement of corticospinal fibers after decussation [2, 5]. Our patient's presentation aligns well with Opalski syndrome from a right medullary infarct with ipsilateral hemiparesis. Similarly, Dhamoon et al. also reported ipsilateral hemiplegia with Wallenberg syndrome, reinforcing the concept that lesion location relative to the decussation determines laterality of weakness [3]. Our case adds to this limited literature, illustrating the occlusion of the vertebral artery can result in recurrent ischemia at the medulla–cervical junction. In our case, the CTA demonstrated a known right vertebral artery occlusion, which led to the right medullary infarct extending below the pyramidal decussation to involve the already‐crossed corticospinal tract fibers. This area is of anatomical importance as the vertebral artery's supply transitions to the anterior spinal artery below the medulla–cervical junction.

Vertebral artery occlusion remains a common cause of medullary infarction, often compounded by diffuse atherosclerosis and uncontrolled vascular risk factors such as hypertension and diabetes [4, 6]. Additionally, prior studies also indicate that vertebral artery compression syndromes and vascular anomalies can contribute to recurrent brainstem ischemia, which at times are responsive to conservative management [7]. This case supports the fact that structural vertebral pathology combined with systemic vascular risk factors significantly increases the probability of recurrent ischemic events.

The diagnostic challenge arises because ipsilateral weakness is more commonly attributed to spinal cord or peripheral nerve pathology. However, careful imaging and clinical correlation demonstrated a medullary infarct in our case, which necessitates the suspicion of posterior circulation stroke even with atypical presentations. The integration of advanced MRI sequences with diffusion‐weighted imaging and high‐resolution vessel wall imaging can delineate between atherosclerotic occlusion, dissection, or compression. This case reinforces the importance of correlating imaging with neuroanatomical principles to avoid diagnostic delays.

From the clinical management preview, the patient benefited from aggressive risk factor optimization and antithrombotic therapy. However, there is no standardized treatment algorithm for vertebral artery occlusion with recurrent ischemia at the medulla–cervical junction. Awareness of atypical stroke presentation is critical for clinicians to avoid diagnostic delays.

In conclusion, vertebral artery occlusion can produce medullary infarcts with atypical presentations, such as ipsilateral hemiparesis when the lesion involves the pyramidal decussation. Clinicians should maintain a high index of suspicion for posterior circulation strokes in patients with vascular risk factors and nuanced neurological findings.

## Author Contributions

**Peter Felton:** conceptualization, writing – original draft. **Yugant Khand:** resources, visualization, writing – review and editing. **Mohammad Gharavi:** resources, writing – review and editing. **Ram Saha:** supervision, writing – review and editing.

## Funding

The authors have nothing to report.

## Ethics Statement

As a single‐case report with the patient's signed consent, no ethics review was required.

## Consent

Written informed consent was obtained from the patient for publication of clinical details and accompanying images.

## Conflicts of Interest

The authors declare no conflicts of interest.

## Data Availability

The data that support the findings of this study are available on request from the corresponding author. The data are not publicly available due to privacy or ethical restrictions.
